# The Administration of Oxytocin or Carbetocin at the Time of Cesarean Section Is Not Associated with Changes in the Electrocardiogram

**DOI:** 10.3390/biomedicines13081946

**Published:** 2025-08-09

**Authors:** Edyta Zagrodnik, Marta Górecka, Anna Surówka, Tomasz Machałowski, Małgorzata Szczuko, Maciej Ziętek

**Affiliations:** 1 Clinical Department of Anesthesiology and Intensive Care of Adults and Children, Pomeranian Medical University in Szczecin, 72-010 Police, Poland; edyta.zagrodnik@pum.edu.pl (E.Z.); marta.gorecka@pum.edu.pl (M.G.); 2 Department of Plastic, Endocrine and General Surgery, Pomeranian Medical University in Szczecin, 72-010 Police, Poland; anna.surowka@pum.edu.pl; 3 Department of Perinatology, Obstetrics and Gynecology, Pomeranian Medical University in Szczecin, 72-010 Police, Poland; tomasz.machalowski@pum.edu.pl; 4 Department of Bromatology and Nutritional Diagnostics, Pomeranian Medical University in Szczecin, 71-460 Szczecin, Poland; malgorzata.szczuko@pum.edu.pl

**Keywords:** carbetocin, oxytocin, cesarean section, electrocardiogram

## Abstract

**Background/Objectives:** Oxytocin as well as carbetocin, a synthetic analog of oxytocin with a longer duration of action, can affect the cardiovascular system, which can be recorded in electrocardiographic Holter recordings. The choice of the appropriate dose of oxytocin or carbetocin should take into account potential cardiovascular effects. **Methods:** A total of 70 pregnant women who previously qualified for elective cesarean section and enrolled in the study were divided into two groups. The oxytocin group (OXY) received 5 IU of oxytocin intravenously (i.v.). The carbetocin group (CARBE) received 100 µg of carbetocin intravenously. Both drugs were used alternatively to contract the uterine muscle. Continuous Holter electrocardiograph recording started 30 min before the procedure and continued until about 3 h after the procedure. **Results:** No abnormalities were observed in either of the analyzed groups during intraoperative ECG recordings at either the highest or lowest recorded heart rate values. The incidence of ST-segment-lowering episodes, the depth of the denivelation and its duration did not differ between the groups studied. The incidence of additional ventricular beats was comparable in the OXY and CARBE groups, both before and after the administration of uterotonic drugs. In the CARBE group, no significant changes in MAP were recorded during the entire ST-segment-lowering period. Analysis of heart rate changes during ST lowering showed no differences between the study groups. **Conclusions:** A thorough analysis of perioperative ECG recordings revealed no significant alterations in ECG patterns, neither in response to oxytocin nor carbetocin administration during cesarean sections.

## 1. Introduction

The adaptive changes in the cardiovascular system that occur during pregnancy are the result of the increased metabolic demands of the growing fetus. These changes are also the result of increased vascularization and volume of blood flow within the uterus and placenta. Among the observed changes, the most substantial are associated with an increase in circulating blood volume, stroke volume, cardiac minute volume and acceleration of heart rate. The aforementioned changes have a demonstrable impact on the pregnant woman’s electrocardiogram (ECG) recording. When conducting such an analysis, it is imperative to take into account the alterations in the position of the heart resulting from the elevation of the diaphragm, as well as the deviation of the heart’s electrical axis to the left by approximately 15 degrees. This deviation is attributed to the upward shift in the heart and forward rotation. The presence of small Q-waves and T-wave inversion in right-sided precordial leads is a relatively frequent occurrence.

During pregnancy, due to dilatation of the peripheral vessels, the heart rate may be accelerated, and many pregnant women have accessory beats. Consequently, the cardiac positional alteration results in the compression of the right ventricle and the subsequent compression of the large vessels [1]. Cardiovascular disease poses a considerable threat to pregnancies in the United States, affecting 1% to 4% of pregnancies on an annual basis. Palpitations and arrhythmias during pregnancy are prevalent and may give rise to concerns regarding the well-being of both the mother and the fetus [2]. Arrhythmias are among the five major risk factors for cardiac complications in pregnant women. It has been demonstrated that there is an elevated risk of primary arrhythmias or exacerbation of pre-existing symptoms during pregnancy. Notwithstanding an increase in heart rate of 25%, the probability of developing severe arrhythmias remains low. Ectopic beats are generally well tolerated and clinically benign. The occurrence of complex ventricular arrhythmias and cardiac decompensation is sometimes precipitated by volume overload, a characteristic of pregnancy [3]. The incidence of ventricular tachycardia (VT) in women of childbearing age is low. In the absence of concomitant organic heart disease, this condition typically arises from elevated catecholamines or augmented cardiac sensitivity during pregnancy, a phenomenon attributable to increased adrenergic receptor density. Premature atrial contractions observed in pregnant women are generally well tolerated. Atrial fibrillation and atrial flutter are rare among pregnant women [2]. Their occurrence during pregnancy is commonly linked to metabolic disorders, including hyperthyroidism or dyselectrolitemia, and congenital heart defects. The pharmaceuticals utilized during cesarean sections to modulate uterine contractile function are crucial for the prevention of obstetric hemorrhage. However, these drugs also have the potential to affect the cardiovascular system [2]. The World Health Organization (WHO) has recommended the administration of oxytocin, a neurohormonal agent that has been demonstrated to induce various physiological responses in humans [4]. This medication has a short half-life and starts working as soon as it reaches the uterine muscle. The side effects of intravenous oxytocin are well known, including heart rate acceleration, low blood pressure (by dilating the vessels) and changes to the electrocardiogram (EKG), which can be dangerous for high-risk patients [5]. The responses to oxytocin administration, including vasodilation, resulting in hypotension [6,7], tachycardia, a decrease in peripheral resistance, signs of myocardial ischemia [8] and an increase in cardiac minute volume, are contingent on the administered dose [9,10]. Oxytocin’s short-term effects may require repeated dosages, which could raise the chance that the same adverse effects will recur [10]. By activating oxytocin receptors in the uterine muscle, oxytocin affects the uterus. Through phospholipase C and the release of inositol triphosphate, it triggers the direct contraction of the uterine muscle, which results in the intracellular release of calcium. It permits the start of labor by increasing the endometrium’s production of prostaglandins. A synthetic analog of oxytocin, carbetocin [1-deamino-1-carbo-2-tyrosine(*O*-methyl)-oxytocin], also an effect of the oxytocin receptor, exhibits a prolonged binding time to the receptor and an extended duration of biological activity, resulting in significant cardiovascular parameter alterations [9,10,11].

Despite the utilization of these pharmaceutical agents in routine clinical practice, the extant literature continues to be deficient in providing precise data that would facilitate a comprehensive comparison of the incidence and severity of adverse cardiovascular sequelae that may ensue subsequent to their administration during cesarean section [11]. The purpose of this study was to compare the effects of oxytocin and carbetocin on the characteristics of Holter-derived electrocardiograph recordings in pregnant women undergoing cesarean section. The additional aim was to obtain direct comparative data regarding safety and effectiveness and to support clinical decision-making based on evidence for the best possible postpartum hemorrhage prevention. There is a big lack of any evidence-based comparison data of both medications (oxytocin and carbetocin), especially prospective studies like ours, which means that the current study is highly necessary.

## 2. Materials and Methods

### 2.1. The Characteristics of the Study Group

This study’s population comprised 70 healthy patients with uncomplicated singleton pregnancies. All of the pregnant women included in the study were term pregnancies between 37 and 41 weeks (mean 38.5 ± 1.4) of gestational age (Table 1).

All cesarean sections were elective surgeries. Excluded from the study were women in whom indications for cesarean section were made during ongoing labor. Also excluded from the study were pregnant women in whom the course of pregnancy was complicated by heart disease, thyroid disorders, diabetes, hypertension, iron deficiency anemia, bronchial asthma, kidney disease, electrolyte disorders (Na^+^, K^+^) and epilepsy.

The peripheral intravenous route for the procedure was secured by a venflon-type cannula 1.3 mm in diameter (16 GA). Intravenous infusion of 500 mL of multi-electrolyte fluid (isotonic multi-electrolyte saline, Fresenius Kabi, Kutno, Poland) occurred within 30 min before the start of anesthesia. Prophylaxis of aspiration pneumonia included oral administration of 30 mL of 0.3 mol/L Sodium Citrate and intravenous administration of 200 mg of Cimetidine (Cimetidine, Jelfa, Poland). In order to standardize the groups, all parturients were anesthetized subarachnoidly.

The subjects were divided into two groups based on the type of uterotonic drug used during the cesarean section procedure. The two study groups did not differ in their indications for cesarean section (Table 2). The OXY group comprised 34 pregnant women who underwent cesarean section on an even-numbered day of the month and received 5 IU of intravenous oxytocin (Oxytocin Grindex, Joint Stock Company GRINDEX, Riga, Latvia) to induce uterine contraction. The CARBE group included 36 pregnant women who were administered 100 µg of carbetocin intravenously (Pabal, Ferring Pharmateucicals, Kiel, Germany) on an odd day of the month during the procedure. The administration of uterotonic drugs occurred 20 s after the birth of the infant, at which time the umbilical cord was also clamped.

During the cesarean section procedure, estimation of intraoperative blood loss was similar in both OXY and CARBE groups and showed no significant differences. The average blood loss, subjectively estimated by the operator during the procedure, was 500 mL.

### 2.2. Method of Carrying Out Measurements

Continuous electrocardiogram recording was initiated 30 min prior to the procedure, subsequent to thorough patient education and the acquisition of written consent. Routine intraoperative and postoperative monitoring, conducted in accordance with current standards, did not affect Holter ECG recording.

The Cardioscan 10-type recorder (manufactured by Oxford Pol, Łódź, Poland) was utilized to record electrocardiographic (EKG) data employing the Holter method. In a system of seven electrodes positioned in the standard configuration, electrocardiographic recordings were obtained using the following three-lead configuration: channel 1—CM-5 (V5, respectively), channel 2—CS-1 (V1) and channel 3—IS (III), with a shift of 25 mm/s and a gain of 1 mV/cm. Continuous electrocardiogram (ECG) recordings were subjected to analysis at two distinct time intervals. The initial period, from the moment the electrocardiogram (EKG) recorder was connected in the antepartum room until the uterine incision, was documented. The second period, which commenced at the time of administration of oxytocin or carbetocin and continued until the conclusion of observation, encompassed a duration of approximately three hours following the procedure, until the central block subsided. Following the completion of the recording, a manual analysis was conducted. This analysis entailed the removal of artifacts and the verification of the systematization of beats (dominant, ventricular). These actions enabled the classification of arrhythmias and the assessment of the occurrence and magnitude of ST segment depression in all leads.

The electrophysiological parameters that were the focus of this study included the minimum, maximum and average rate of the dominant heartbeat; the number and quality of additional ventricular beats; and the number and quality of ST segment depression or elevation. The values of ST segment depression and elevation, defined as significant, as well as the classification of arrhythmias, were carried out in accordance with the current recommendations of the Polish Society of Cardiology. The electrophysiological parameters obtained were then analyzed in relation to data from prior to the procedure.

### 2.3. Statistical Analysis

Statistical analysis was performed using Statistica PL v. 9.0, a StatSoft Tulsa, Oklahoma, United States package. Continuous variables were presented as the arithmetic mean and standard deviation, while qualitative variables were presented as counts and relevant percentages. The normality of continuous variable distributions was verified using the Shapiro–Wilk method. The two randomly selected study groups were initially compared for general characteristics using Student’s *t*-test for arithmetic means and Pearson’s Chi-squared test for percentages. From the administration of the uterine contraction drug until the end of the procedure, relevant parameters were measured first every minute and then every three minutes. ANOVA for repeated measures and Fisher’s NIR test (post hoc test) were then applied in a group × time arrangement. The significance level was set at *p* < 0.05.

## 3. Results

A comparison of the study groups showed no differences in the mean duration of Holter ECG monitoring (Table 3).

A comparative analysis of the minimum (HR_min_) and maximum (HR_max_) heart rate values recorded during Holter electrocardiography was conducted, encompassing both preoperative and intraoperative phases, following the administration of uterotonic medications. This analysis revealed no significant differences between the study groups (Table 4).

**Figure 1:**
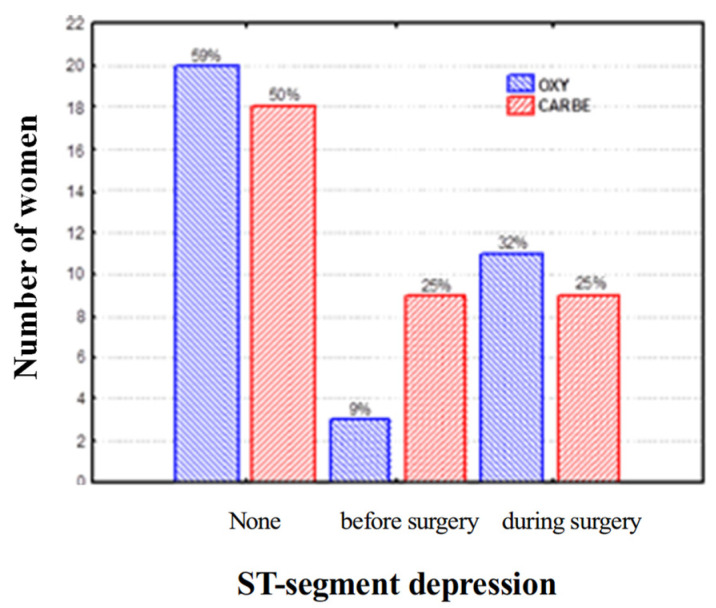
Frequency of ST segment depression during Holter ECG recording.

With regard to the occurrence of ST segment depression, including the preoperative and intraoperative periods, after the administration of uterotonic drugs, the study groups did not differ (Figure 1). No pregnant woman exhibited ST segment depression in both analyzed periods, i.e., before and after uterotonic drug administration.

**Figure 2:**
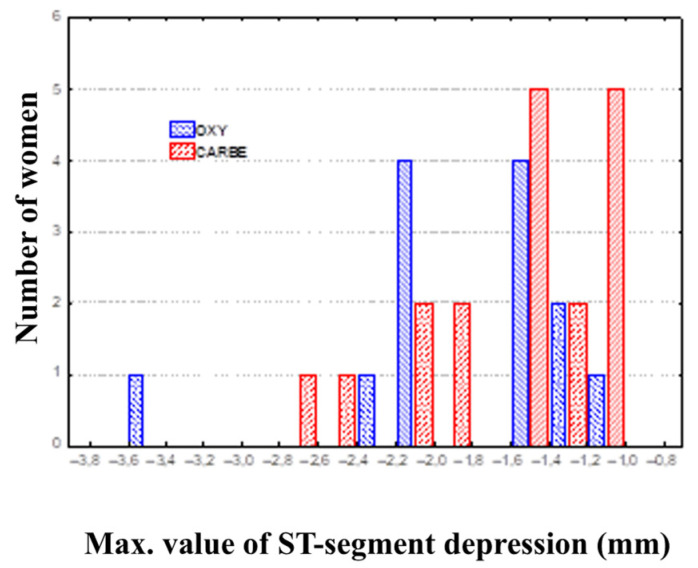
Maximum value of ST segment depression in continuous ECG recording using the Holter method.

**Figure 3:**
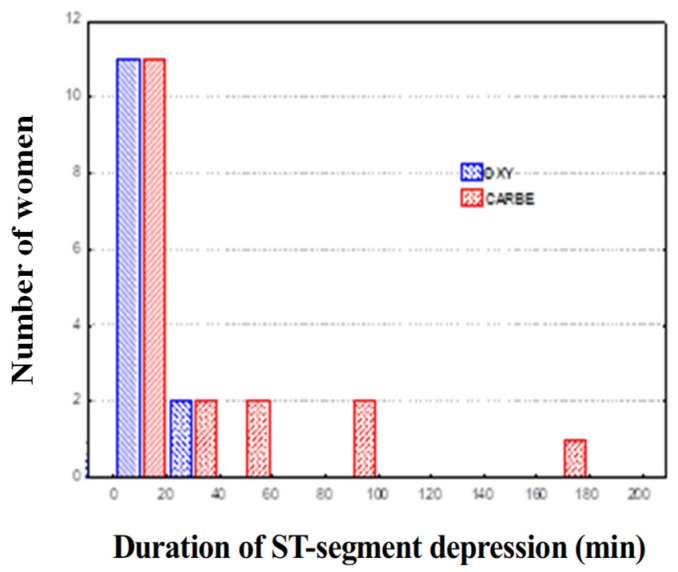
Duration of ST segment depression during Holter ECG recording.

**Figure 4:**
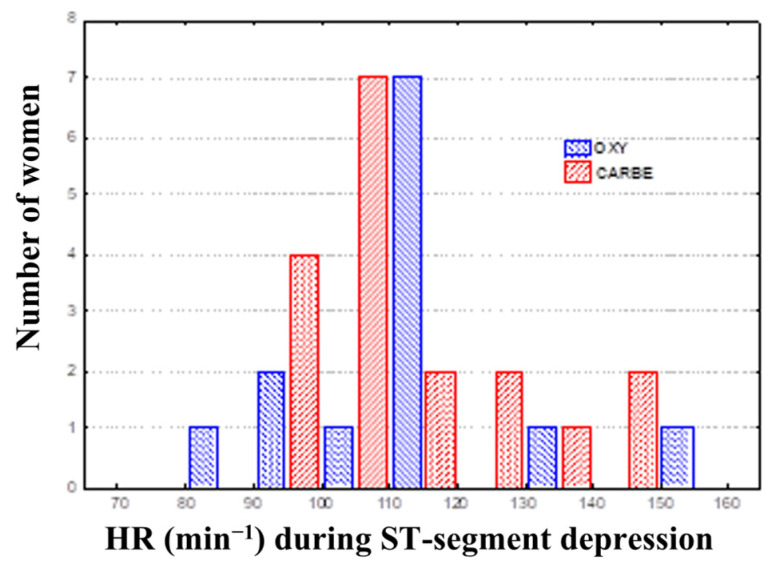
Heart rate during ST segment depression during Holter ECG recording.

Comparing the data for the OXY and CARBE groups, including maximum ST segment depression values, the ST segment depression duration and heart rates during ST segment depression in the preoperative period and after uterotonic drug treatment showed no significant differences between the groups (Figure 2, Figure 3 and Figure 4).

**Figure 5:**
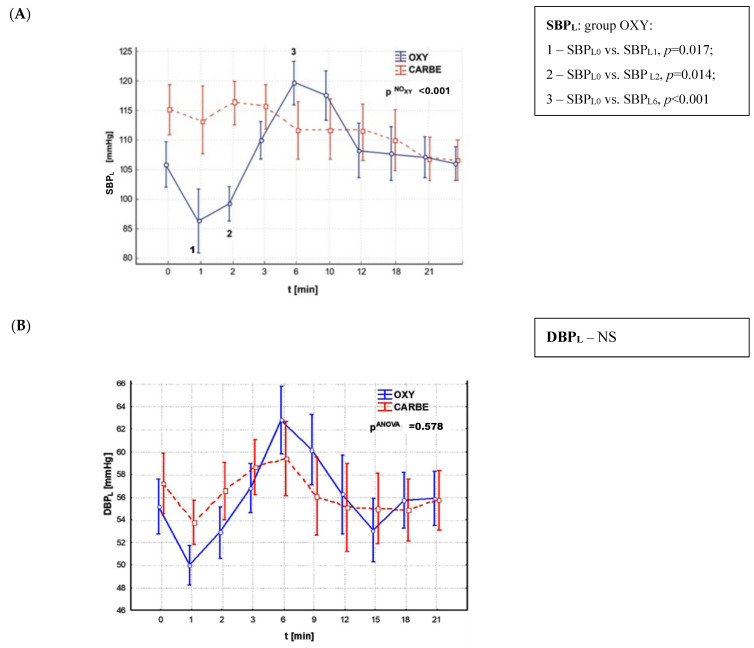

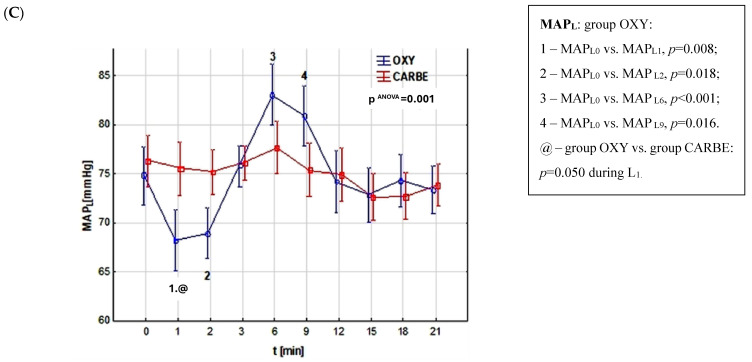
Blood pressure values: (**A**) systolic (SBP_L_), (**B**) diastolic (DBP_L_) and (**C**) mean arterial pressure (MAP_L_) during ST segment depression after uterotonic drug administration.

**Figure 6:**
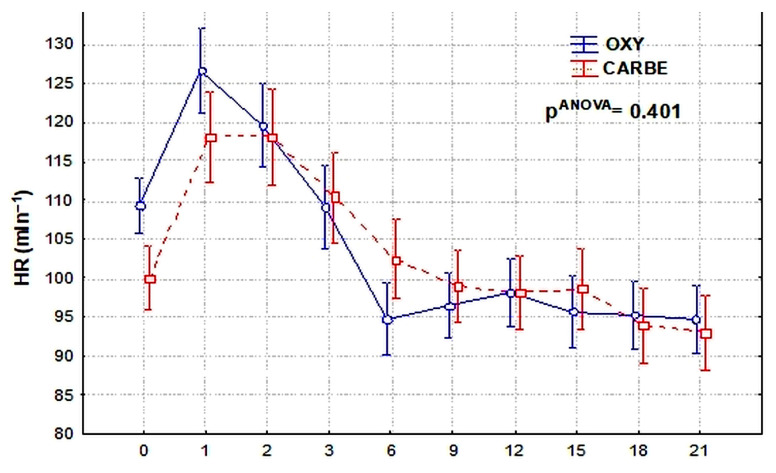
Heart rate (HR_L_) during ST segment depression after uterotonics.

An important aspect of this study was to compare changes in systolic, diastolic and mean arterial pressure, as well as heart rate, during ST segment depression after the administration of oxytocin and carboprost (Figure 5 and Figure 6).

A comparison of systolic blood pressure (SBP) values during ST segment lowering revealed significant differences in the OXY group, where a substantial decrease in SBP was observed one minute after oxytocin administration (*p* = 0.017) which persisted during the subsequent minute (*p* = 0.014) and subsequently exhibited a significant increase after the sixth minute following drug administration (*p* = 0.001). In contrast, the CARBE group exhibited no substantial changes in SBP. The study groups did not differ in systolic blood pressure values during ST segment depression.

A subsequent analysis of the values of diastolic blood pressure during ST segment lowering, recorded at different periods of the procedure, showed no statistically significant differences both within and between the study groups.

With regard to alterations in mean arterial pressure during ST segment lowering, statistical significance was recorded exclusively in the OXY group, in which a decrease in MAP was observed one minute after drug administration (*p* = 0.008), which was sustained over the subsequent minute (*p* = 0.018), followed by a significant increase six minutes after oxytocin administration (*p* = 0.001). No significant changes in mean arterial pressure (MAP) were recorded in the CARBE group during the entire observation period. The study groups exhibited significant variations in mean arterial pressure (MAP) values following the initial minute of oxytocin and carbetocin administration. A pronounced decrease in MAP was observed in the OXY group in comparison to the CARBE group (*p* = 0.05).

The evaluation of the heart rate values recorded during ST lowering at different periods of the cesarean section procedure showed no statistically significant differences between the study groups.

During Holter ECG monitoring, additional ventricular contractions occurring in the subjects in both groups were recorded. Comparison of the groups in this regard showed no statistical significance (*p* = 0.676). The incidence of ventricular beats in the OXY and CARBE groups both before the administration of the uterotonic drug (43.5% vs. 46%) and after the administration of oxytocin and carbetocin (56% vs. 54%) was comparable.

## 4. Discussion

The utilization of pharmaceutical agents that modulate uterine contractility is imperative during a cesarean section procedure. The pharmaceuticals in question have been demonstrated to exert an effect on a number of parameters within the cardiovascular system. This, when considered in conjunction with the adaptive changes that occur during pregnancy, may possess significant clinical importance, particularly in women who are also afflicted with concomitant heart disease.

In a prospective, randomized, double-blind study using non-invasive pulse wave analysis, Rabow et al. analyzed the cardiovascular effects of oxytocin and carbetocin during cesarean section. The authors concluded that the drugs had the same vasodilatory and hypotensive effects. Oxytocin, but not carbetocin, caused a decrease in HR after 1 min and a sustained decrease in left ventricular ejection time. None of the drugs caused a change in ST score, SaO2 or subjective cardiac symptoms [12]. The information obtained in our study included data on heart rate, quantity and quality of cardiac arrhythmias and ST segment evaluation. A thorough analysis was conducted on the highest and lowest recorded heart rates, with the objective of determining whether there was a statistically significant difference between the groups. However, the analysis yielded no statistically significant difference. A comparison of the two groups in terms of ST segment rate and morphology demonstrated that there were no statistically significant differences between the OXY and CARBE groups. With regard to the alterations in systolic pressure and mean arterial pressure during intraoperative ST segment depression, statistical significance was observed following the administration of oxytocin. Furthermore, diastolic pressure values during ST segment depression were found to be similar between the two groups. The incidence of additional ventricular beats was found to be equally prevalent in both the OXY and CARBE groups.

The results obtained in the present study, in terms of changes in perioperative ECG recordings, demonstrate partial consistency with the results of the work of other investigators. The literature describes the physiological changes associated with the pregnancy period, particularly in the latter stages, which can result in abnormalities in the pregnant woman’s electrocardiogram recordings [13]. ST segment abnormalities are also described as physiological changes that occur during the late period of pregnancy [14].

The hypothesis that changes in the electrocardiogram recording in pregnant women may suggest myocardial ischemia has been confirmed by an extensive analysis by Mathew et al. [15]. The present study recorded ST segment depression in continuous Holter ECG recordings in women undergoing cesarean section. This occurred with equal frequency after subarachnoid (17%) and general anesthesia (18%). As demonstrated in the aforementioned study, the proportion of pregnant women who exhibited documented ST segment depression was recorded at 25%. This reduction in tone occurred most often during the intraoperative period and up to 30 min after delivery. In 10% of cases, ST segment depression was accompanied by a significant increase in heart rate. The authors of the study report that no incidents of ST segment elevation were observed in any of the pregnant women participating in the study. In the study by Palmer et al. [16], the percentage of pregnant women with recorded electrocardiographic changes during cesarean section was higher at 37, with 35 of the 44 patients with ECG changes having ST segment depression, suggestive of myocardial ischemia. This was accompanied by a moderate increase in heart rate, which was temporally associated with a decrease in systolic and diastolic blood pressure.

A randomized study by Rabow et al. analyzed 61 healthy pregnant women undergoing elective cesarean section under subarachnoid anesthesia [12]. The subjects were randomly divided into two groups depending on the drug administered: Group I received an intravenous bolus of five units (8.3 µg) of oxytocin, and Group II received 100 µg of carbetocin after delivery. The present study examined heart rate, mean arterial blood pressure, ST segment in ECG, oxygen saturation (SaO_2_) and photoplethysmographic variables of digital pulse wave analysis. The parameters were recorded prior to and following administration of the drug, as well as 1, 5, 20 and 60 min thereafter. In conclusion, the authors observed in the G 1 group, which received oxytocin, a decrease in heart rate (HR) after one minute and a sustained decrease in left ventricular ejection time compared to the group receiving carbetocin. The investigation revealed that neither pharmaceutical intervention resulted in alterations to the electrocardiogram ST index, oxygen saturation levels or subjective cardiac symptoms. Single doses of oxytocin and carbetocin were found to have comparable vasodilator effects on vascular tone. The administration of oxytocin was followed by a transient negative chronotropic effect and a negative inotropic effect [12].

McLintic et al. analyzed the morphology of changes in continuous Holter ECG recordings in pregnant women undergoing cesarean sections. They recorded ST segment depression suggestive of myocardial ischemia in 16 of the 25 women studied (64%). During ST segment lowering, the mean heart rate in these pregnant women was significantly higher compared to subjects with no ST changes (127.9 min^−1^ vs. 111.7 min^−1^). The study’s authors demonstrated that there was no statistically significant change in systolic pressure during ST segment depression [17]. In a comprehensive study by Zakowski et al. [18], the authors analyzed continuous Holter electrocardiographic recordings in pregnant women during cesarean sections. This analysis was consistent with the findings of other studies on the subject, which also reported ST segment depression.

The study by Moran et al. described cases of myocardial ischemia (affecting 7.69% of patients) that occurred alongside ECG changes observed during elective cesarean sections, as well as episodes of significant postoperative ST segment changes [19]. ST segment depression occurring during CS is associated with a hyperkinetic myocardial contractile state, which is characterized by an abnormally rapid heart rate [20]. ECG changes occur in healthy patients undergoing routine elective cesarean section at similar rates after both narrow- and standard-bandwidth ECG filtering modes for assessing ECG-detected ischemic changes [21]. ST segment depression is often observed in women undergoing cesarean sections with regional anesthesia. However, myocardial ischemia seems an unlikely cause of such depression in this healthy population [22,23,24]. It is noteworthy that the vast majority of these alterations, amounting to over 98%, transpired during the period between the induction of anesthesia and the conclusion of the procedure. ST segment lowering was accompanied by an increase in heart rate in 10% of cases. A further study analyzed a group of 40 healthy singleton pregnant women, ranging in age from 18 to 50 years and with a gestational age of at least 36 weeks, who underwent elective cesarean sections. Participants were randomly assigned to a group receiving oxytocin 2.5 IU or carbetocin 100 μg immediately after delivery. The QTc interval, ST segment depression and relative increase in heart rate were recorded from the commencement of the study drug until 10 min after delivery. The findings did not demonstrate statistical disparities in the study groups with regard to QTc (*p* = 0.13) and ST segment depression (*p* = 0.11) in ECG recordings [25]. The results presented in the study are partially comparable with the results described by the authors of the works available in the literature. In the present study, as in the aforementioned studies of Mathew et al. [15], Palmer et al. [16] and Zakowski et al. [18], a decrease in the ST segment in continuous ECG recordings was observed more frequently during the intraoperative period. In a manner analogous to the findings of the Palmer study, this investigation revealed a substantial decline in systolic blood pressure following the administration of oxytocin, concomitant with a reduction in ST segment.

In consideration of the fluctuations in diastolic blood pressure during ST segment depression, the findings of the present study stand in contrast to those attained by Palmer [16]. Palmer’s observations documented a substantial decline in this parameter subsequent to the administration of oxytocin, a phenomenon that was not discernible in the present study. The moderate increase in heart rate that was observed during the course of the study, and which accompanied the ST segment depression, differed from the results that were published by Eisenach et al. [22]. In his work, he documented a decline in the ST segment in 33% of patients following spinal anesthesia, with this decline occurring within a time frame of 5–30 min. Furthermore, in pregnant women with a confirmed ST segment depression, he observed a significantly higher heart rate compared to those without ST segment depression (117 ± 7 vs. 94 ± 6 bpm), as well as the need for larger doses of ephedrine in this group (25 ± 3 mg vs. 7 ± 3 mg). This was not observed in the present study. In order to comprehend the reasons for the frequent occurrence of ST segment depression in healthy pregnant women during cesarean section, it is imperative to consider the anxiety experienced by these individuals. Potential contributing factors include hyperventilation, ionic disturbances or changes in sympathetic nervous system tone resulting from anesthesia or the positioning of the pregnant woman during the procedure [24,26].

As posited by certain researchers, in the aftermath of spinal anesthesia, in conjunction with existing sympathetic block, the occurrence of sudden hypervolemia can be attributed to prehydration of the pregnant woman and autotransfusion of blood from the contracted uterus to the vascular bed of the placenta [16,27]. This phenomenon results in a transient increase in end-diastolic volume and pressure, thereby augmenting the workload of the heart and the demand for oxygen. Simultaneously, the occurrence of a sympathetic blockade, resulting in a reduction in diastolic pressure, has the potential to compromise myocardial perfusion. This phenomenon, when compounded by an escalated oxygen demand and a diminished oxygen supply, may precipitate myocardial ischemia, as evidenced by the ECG. Palmer et al. [16] do not preclude the possibility of vasoconstriction in the pathogenesis of ST segment changes.

It is imperative to consider the increased prevalence of lower ST segment elevation during the intraoperative period, given the potential impact of pharmaceutical agents employed for uterine contraction during this phase. In the extant literature, there is a description of episodes of myocardial infarction associated with the administration of oxytocin [28,29]. The intravenous administration of oxytocin has been demonstrated to induce vasodilation of the intramuscular arteries, leading to a reduction in both the systolic and diastolic pressures. Of particular significance is the potential impact on the coronary perfusion, which may be compromised as a consequence. The combination of such an action with physiological anemia in pregnant women has been shown to result in impaired oxygen delivery to the level of the organ system that is responsible for the body’s current oxygen requirements. The study by Moran et al. sought to identify other potential causative factors for symptoms indicative of myocardial infarction in pregnant women [19]. The present study investigated the impact of three factors on electrocardiographic readings: hypotension, tachycardia and an administration of epinephrine. The study found no evidence of a correlation between the administration of oxytocin and the occurrence of arrhythmias in the ECG. Yaliwal RG. et al. reported similar outcomes in a randomized study involving 212 pregnant women in the third trimester of pregnancy who underwent cesarean section [30]. The women who were part of the study were divided into two groups. A comparison was made between the two groups, with Group I receiving 50 micrograms of carbentocin IV and Group II receiving 100 micrograms of carbentocin IV. The primary outcome measures were heart rate and blood pressure. The presence of blood samples containing oxygen, alterations in electrocardiographic readings and preoperative (12 h after cesarean section) levels of troponin I, which is highly sensitive, were all detected. No significant statistical differences were observed between the groups in relation to the investigated cardiovascular parameters (*p* > 0.05). The study revealed that no adverse cardiovascular effects were observed in any of the participants. Furthermore, no significant differences were found in cardiovascular outcomes between the study groups [30]. The extant literature on the subject indicates the existence of a multifactorial process with the potential to induce the manifestation of ventricular tachycardia in patients with congestive heart failure, suggestive of myocardial infarction.

The following limitations must be noted: The primary constraint of our study is the modest sample size, which, in the event of a more substantial cohort of women, has the potential to impact the study’s findings.

The extension of the patient population under consideration in this study would facilitate the identification of infrequent adverse events (e.g., rare cardiac arrhythmias), thereby enhancing the study’s significance, both in terms of its theoretical underpinnings and its practical applications in the clinical realm.

This study’s design, which focused exclusively on healthy pregnant women with low-risk pregnancies (i.e., without concomitant heart disease), enabled the creation of a homogeneous study group. This methodological approach allowed for objective comparison of the effects of both drugs. There is a compelling rationale for conducting the aforementioned study in women suffering from heart disease prior to pregnancy or multiorgan disorders that occurred during pregnancy (e.g., preeclampsia, hypertension, additional ventricular or supraventricular contractions of the heart) in the future.

A further limitation of this study is the non-determination of concentrations of cardiac biomarkers, such as cardiac troponin T, in the blood. Increased values of cardiac troponin T indicate myocardial damage and are particularly important for rapid confirmation or exclusion of myocardial infarction.

It is important to note that not all women possess analogous metabolic pathways that are implicated in the inactivation of the pharmaceuticals under investigation. Due to individual characteristics, these pathways can modify the actual impact of the pharmaceuticals on ECG. The varying emotional states of the women participating in the study, which are associated with elevated stress levels and the activation of various hormones, may have also contributed to the observed disruption in the study’s results.

## 5. Conclusions

This study revealed that no adverse cardiovascular effects were observed in any of the participants. The heart rate, quantity and quality of cardiac arrhythmias and ST segment evaluation after administration of 100 µg of carbetocin intravenously or 5 IU of intravenous oxytocin did not show any differences; therefore there is no indication for the use of a specific drug in the doses discussed.

## Figures and Tables

**Table 1:** Medical and demographic data of pregnant OXY and CARBE groups.

Parameters	Group OXY (n = 34)			Group CARBE (n = 36)			*p*-Value
	Mean ± SD	Min.–Max.	Median	Mean ± SD	Min.–Max.	Median	
Age (years)	29.7 ± 5.3	20–39	30.0	29.3 ± 4.9	20–46	29.5	NS
Gestational age (weeks)	38.5 ± 1.4	37–41	39.0	38.9 ± 0.9	37–40	39.0	NS
Body weight (kg)	76.5 ± 9.6	55–97	76.5	79.8 ± 12.7	57–110	78.0	NS
Height (cm)	165.3 ± 5.0	156–176	165.5	166.0 ± 4.1	158–182	164.5	NS
BMI (kg/m^2^)	28.0 ± 3.4	22.6–37.8	28.1	28.9 ± 4.1	20.1–37.0	28.3	NS

Student’s *t*-test, NS: non-statistical significance, SD: standard deviation.

**Table 2:** Indications for cesarean section surgery.

Indications for Cesarean Section Surgery	Group OXY (n = 34)		Group CARBE (n = 36)	
	n	(%)	n	(%)
Thin lower uterine segment after cesarean section	16	(47)	15	(42)
Pelvic position of the fetus	5	(15)	9	(25)
Macrosomia of the fetus	3	(8.5)	3	(8)
Fetal–pelvic disproportion	3	(8.5)	3	(8)
Ophthalmologic indications	2	(6)	4	(11)
Tokophobia	2	(6)	1	(3)
Polyhydramnios	2	(6)	0	(0)
Ahydramnios	1	(3)	1	(3)

**Table 3:** Duration of continuous ECG recording in the OXY and CARBE groups.

Variable	OXY Group (n = 34)		CARBE Group (n = 36)		*p*-Value
	Mean ± SD	Median	Mean ± SD	Median	
Duration of ECG monitoring (min)	273 ± 102	272	266 ± 114	231	NS

Student’s *t*-test; NS: non-significant.

**Table 4:** The following data represent the lowest (HR_min_) and highest heart rate (HR_max_) as recorded in continuous Holter electrocardiography in the OXY and CARBE groups.

Variable		OXY Group (n = 34)	CARBE Group (n = 36)	*p*-Value
		Mean ± SD	Mean ± SD	
HR_max_ (min^−1^)	before administration of uterotonic drug	135.1 ± 11.2	140.4 ± 13.1	NS
	after administration of uterotonic drug	139.6 ± 11.7	146.4 ± 12.9	NS
HR_min_ (min^−1^)	before administration of uterotonic drug	59.2 ± 8.1	62.3 ± 9.2	NS
	after administration of uterotonic drug	57.4 ±7.2	59.2 ± 8.1	NS

Student’s *t*-test; NS: non-significant.

## Data Availability

Data are available on request from the first author.
